# Author-based journal selection system that helps authors save time in article submission

**DOI:** 10.12669/pjms.341.14074

**Published:** 2018

**Authors:** Onur Ozturk, Fatih Ileri

**Affiliations:** 1 Onur Ozturk, Family Medicine Specialist/ Sexual Therapist, Department of Family Medicine, Asarcik Meydan Family Healthcare Center, Samsun, Turkey; 2 Fatih Ileri, Senior SW Design Engineer, Department of Space Systems, Turkish Aerospace Industries Inc., Ankara, Turkey

**Keywords:** Article, Submission, Author, Journal

## Abstract

Submission to journals takes a lot of time and format related submission requirements vary greatly from one journal to another. Lack of time and motivation in academia reduces scientific outputs and demotivates researchers. Author-based journal selection system (ABJSS) is a platform for pooling manuscripts conceived to minimize the time spent for manuscript submission and to increase scientific output. The system will provide two types of account: “Author” and “Journal Administrator”. Each account type will have its own abilities and permissions.

The ABJJS system is an ongoing project that will be designed in cooperation with IT experts and academicians and it will be presented to the scientific world as soon as it secures sufficient support.

## INTRODUCTION

Original works, reviews, case reports and letters are obviously important for any evident-based academic activity. In addition to the labor-intensive and challenging nature of writing, authors also face several problems during the publication period. Submission to journals takes a lot of time and format related submission requirements vary greatly from one journal to another. The submission process may take several days, and the evaluation process by the journal between several months and years while; overall the time between admission and publication can take a few months or even 1-2 years. This challenging and time-consuming process may lead to frustration and low performance of authors most of whom even give up the submission process altogether.

Lack of time and motivation in academia reduces scientific outputs and demotivates researchers. Unfortunately, this problem has not been addressed in the scientific literature because the authors have not tried to solve this problem enough. Even so, even when we send this letter, we could not find a suitable reference.

## METHODS

Author-based journal selection system (ABJSS) is a platform for pooling manuscripts conceived to minimize the time spent for manuscript submission and to increase scientific output. The aim is to help volunteer member journals view submitted manuscripts and inform authors about required revisions and estimated time for publication. Authors will be able to upload their abstracts and keywords to the ABJSS. The full text will be available only upon a direct demand by a journal and an authorization by the author. Just as authors send journals an undersigned declaration of conflict of interests, accompanied to their submitted journals, journals will guarantee that they will never use the manuscript data and otherwise they will have temporary publication ban due to ethical violation.

Authors may receive requests for revision or delay by several journal, but they can accept only one. If an author accepts a proposal from a journal, s/he cannot receive proposals from any other journal until s/he withdraws his/her article as the system does not allow him/her to do it. This will solve authors' problem of lack of time and motivation while preventing journals from becoming victims.

**Fig. 1:**
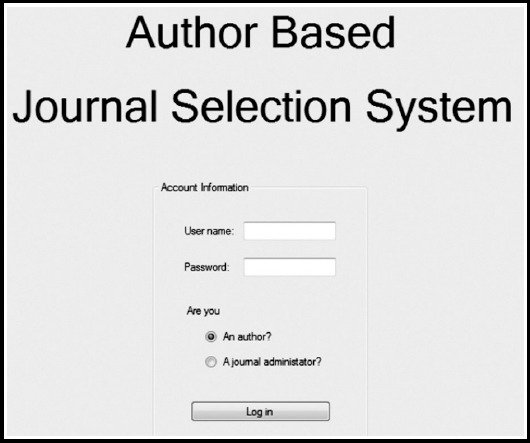
The login screen.

**Fig. 2:**
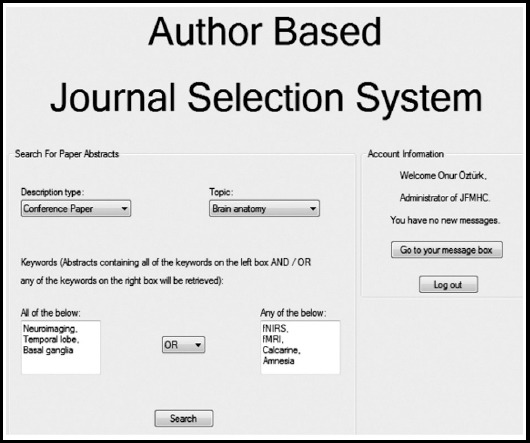
An abstract search screen.

Sample images of the designed project are shown in this write-up. In the Fig.1, the login screen is shown. In the Fig.2, an abstract search screen is displayed, which the journal administrator will encounter when logging into the system. These sample images will be much more varied and detailed. In this process, we are open to the proposals of the scientific world. Technical overview of ABJSS is below:

### Usage

The system will provide two types of account: “Author” and “Journal Administrator”. Each account type will have its own abilities and permissions.

Author will be able to upload abstracts and evaluate full-text view requests from Journal Administrators. Once an Author accepts a request from a Journal Administrator, he/she will not be able to contact with other Journal Administrators through the system related to that specific scientific paper.

Journal Administrator will be able to apply advanced search for abstracts such that the system will provide description type and topic selection from a wide variety of options. In addition, the system will let the Journal Administrator to generate complex search filters by involving AND and OR operations between keywords.

### Security

System security will be handled in such a way that the paper abstracts and account information (including the messsages between the Authors and the Journal Administrators) will be kept in system by encrypting the texts with 128bits AES encryption methodology.

### Redundancy

Redundancy will be provided through periodically generated database images through the SVN version control tool. These database images will be kept in an offline location which will be independent from the system server.

## CONCLUSION

The ABJJS system is an ongoing project that will be designed in cooperation with IT experts and academicians and it will be presented to the scientific world as soon as it secures sufficient support. It is planned to be used initially for the medical literature; but its integration with other fields will be also highly useful.

### Author's Contributor

***Onur Ozturk:*** Conceptualized and designed the project.

***Fatih Ileri:*** Drafted the initial manuscript, critically reviewed the manuscript, and approved the final manuscript as submitted.

